# Construction and Evaluation of *V. cholerae* O139 Mutant, VCUSM21P, as a Safe Live Attenuated Cholera Vaccine

**DOI:** 10.1371/journal.pone.0081817

**Published:** 2014-02-05

**Authors:** Chandrika Murugaiah, Nik Zuraina Nik Mohd Noor, Shyamoli Mustafa, Ravichandran Manickam, Lalitha Pattabhiraman

**Affiliations:** 1 School of Health Sciences, Universiti Sains Malaysia, Kubang Kerian, Kelantan, Malaysia; 2 Newcastle University Medicine Malaysia (NUMed), Nusajaya, Johor, Malaysia; 3 Department of Medical Microbiology and Parasitology, School of Medical Sciences, Universiti Sains Malaysia, Kubang Kerian, Kelantan, Malaysia; 4 Department of Department of Biotechnology, AIMST University, Bedong, Kedah Darul Aman, Malaysia; Federal University of Pelotas, Brazil

## Abstract

Cholera is a major infectious disease, affecting millions of lives annually. In endemic areas, implementation of vaccination strategy against cholera is vital. As the use of safer live vaccine that can induce protective immunity against *Vibrio cholerae* O139 infection is a promising approach for immunization, we have designed VCUSM21P, an oral cholera vaccine candidate, which has ctxA that encodes A subunit of ctx and mutated rtxA/C, *ace* and *zot* mutations. VCUSM21P was found not to disassemble the actin of HEp2 cells. It colonized the mice intestine approximately 1 log lower than that of the Wild Type (WT) strain obtained from Hospital Universiti Sains Malaysia. In the ileal loop assay, unlike WT challenge, 1×10^6^ and 1×10^8^ colony forming unit (CFU) of VCUSM21P was not reactogenic in non-immunized rabbits. Whereas, the reactogenicity caused by the WT in rabbits immunized with 1×10^10^ CFU of VCUSM21P was found to be reduced as evidenced by absence of fluid in loops administered with 1×10^2^–1×10^7^ CFU of WT. Oral immunization using 1×10^10^ CFU of VCUSM21P induced both IgA and IgG against Cholera Toxin (CT) and O139 lipopolysaccharides (LPS). The serum vibriocidal antibody titer had a peak rise of 2560 fold on week 4. Following Removable Intestinal Tie Adult Rabbit Diarrhoea (RITARD) experiment, the non-immunized rabbits were found not to be protected against lethal challenge with 1×10^9^ CFU WT, but 100% of immunized rabbits survived the challenge. In the past eleven years, *V. cholerae* O139 induced cholera has not been observed. However, attenuated VCUSM21P vaccine could be used for vaccination program against potentially fatal endemic or emerging cholera caused by *V. cholerae* O139.

## Introduction

*V. cholerae* is the causative agent of cholera which is a life-threatening, acute secretory diarrhoeal disease, a major public health problem that affects indigenous populations [1]. Despite efforts taken to control *V. cholerae* infection [2], [3], the global burden of cholera is high, mostly affecting young children [4]. Though the disease is controllable by clean water supply and sanitation coverage [5], limiting the disease transmission persists as a challenge to most developing countries [5], [6]. An estimated 91,000 people die of cholera in endemic countries annually [7].

Cholera is associated with expression of CT encoded in the ctxAB gene, which is acquired by *V. cholerae* from the filamentous phage CTX [8]. The 27.2-kDa catalytic *ctx*A toxin is responsible for activating the excretory chloride transport in the intestinal crypt cells, while the *ctx*B helps in the internalization of the *ctx*A [9]. Apart from the *ctx*AB toxin, the zonula occludens toxin (*zot*) and accessary cholera enterotoxin (*ace*), located on a 4.5-kb central core region of the CTX genetic element [10]–[13], were suggested to cause probable diarrhoea symptom [11], [14], [15].

The cytotoxic activity of *V. cholerae*, which leads to actin depolymerization of HEp-2 cells in vitro, is caused by RTX toxin [16]. Although cholera is commonly considered to be a noninflammatory secretory disease, RTX toxin produced by *V. cholerae* O1 El Tor was demonstrated to contribute to the severity of acute inflammatory responses [17]. Its operons exist as a four-component type I secretion system (TISS) in the order *rtx*CABD which plays a vital role as an activator and a transporter of the toxin. The GD-rich repeated motif, *rtx*A, is the structural component and also the crucial factor for the cytotoxicity of RTX. *Rtx*A requires *rtx*C, an acyl-carrier-protein-dependent acyl- modification enzyme, for its conversion to active form [18].

Before 1992, cholera pandemic was exclusively caused by O1 serovars. But since 1992, a new non-O1 serovar the *V. cholerae* O139, emerged [19], [20]. Mysteriously, it acquired the ability to overtake its predecessor strains, by establishing several cholera endemics [21]–[23] with comparable clinical severity to El Tor O1 infection [24], [25]. Rapidity of its spread was mainly due to lack of acquired protective immunity in the population against it. *V.cholerae* O139 has been undetected since 2005 [26]. Vaccine candidate killed bivalent (O1 and O139) whole-cell oral cholera vaccine is available [27]. Licensed WHO approved vaccines are available for proection against cholera [3]. *V. cholerae* O139 could become a major global pathogen again and development of new vaccine is required. *V. cholerae* O139 attenuated vaccine strain has already been developed [28]. Oral killed bivalent vaccine (against O1 and O139) is now WHO pre-qualified and has been administered to hundreds of thousands of individuals in Asian Bangladesh [27].

Lethal water-borne infection by *V. cholerae* that had caused a devastating case fatality rate and potential pandemic emphasized the need for adequate clean water supply and sanitation facility [4]. In endemic-prone areas, pursuing the use of vaccine could be an effective alternative option to control cholera cases [3], [4], [29]. A number of important advances in genetic engineering led to a new paradigm in cholera vaccine construction. In this regard, the protection provided by the live attenuated vaccines is of particular interest. The interaction between live attenuated vaccines and immune system is much more effective than with killed, whole cell vaccines [30]. Attenuated O1 vaccines CVD103 HgR, and Peru 15 have been evaluated in humans and are very safe and immunogenic. .A live cholera vaccine against *V. cholerae* presented several challenges [31], including mild diarrhoea experienced by volunteers [32].

In the past several years, investigators at our laboratory attempted to develop several live cholera vaccines against *V. cholerae* O139. Recently, we have constructed a live VCUSM14 auxotroph vaccine candidate, shown to be a potentially efficient attenuated cholera vaccine that secretes enzymatically inactive *ctx*A subunit which is immunogenic yet safe [33]. The 7^th^ amino acid of the *ctx*A which is the arginine has been substituted with lysine (R7K), and the 112^th^ glutamate was substituted with glutamine (E112Q). In this study, we used the VCUSM14 as a parent vaccine for the construction of a new vaccine candidate. A previous attenuated vaccine O139 strain was made and their modifications have advantages over that previously developed vaccine with less reactogenicity. As the virulence factor of *rtx*CABD gene cluster of *V. cholerae* may be associated with residual adverse properties displayed by certain attenuated cholera vaccines [16], the toxin involved in the covalent cross-linking of cellular actin activity, the RTX toxin, has been rendered non-functional in VCUSM14. This safety issue is addressed in order to provide enough assurance for its future use in humans. Besides mutating the *rtx*C/A gene, the *hem*A gene was introduced homogonously onto the chromosome. The resultant vaccine candidate is designated as VCUSM21P. This designed vaccine candidate synthesizes its own δ-aminolevulenic acid (ALA) and encodes the critical, non-toxic *ctx*A immunogen for protection against cholera, and carries *ace*, *zot* and *rtx*C/A mutations. The aim of this study was to modify VCUSM14, which already carried the desired mutations in ctx, ace and zot, by mutating rtx and replacing the mutated hemA by the wild-type allele. There is no disease burden due to *V.cholerae* O139 induced cholera in the last decade. In this investigation, we analyzed the potential use of VCUSM21P as an attenuated vaccine candidate against *V. cholerae* O139.

## Materials and Methods

### Bacterial strains, plasmids and growth media

The details of the description and origin of the bacterial strains and plasmids used in this work are in Table 1. Luria – Bertani (LB) broth was used for both liquid and agar media. Antibiotics were added when appropriate at the following concentrations: polymyxin B (poly), 0.75 µg/mL; ampicillin (amp), 100 µg/ml; Kanamycin (Kan), 50 µg/mL. Autotrophic strains were maintained in Luria-Bertani (LB) medium containing aminolevulenic acid (80 µg/mL) supplementation with appropriate antibiotics. Plasmids, based on the R6K origin of replication which requires the product of the *λpir* gene for replication, were used for conjugal mating. The plasmids were transferred into *E. coli* BW 20767 *λpir* using heat shock method. For counter selection with *sac*B-containing plasmids, LB without NaCl and with 10% sucrose was used.

**Table 1:** Bacterial strains and plasmids used in this study.

Strains or plasmids	Description of strains or plasmids	Source
Strains		
*E. coli* BW 20767 *λpir*	*RP4 2tet: mu-1kan::Tn7integrant leu 63::IS10 rec A1 cre(510 hsdR17 end A1 Zbf-5 uid) (ΔMluI): pir thi*	Gift from Dr. William Metcalf, University of Illinois
WT	*V. cholerae* O139 isolated from a patient in HUSM	Hospital University Sains Malaysia
VCUSM14	*V. cholerae* O139 ALA auxotroph derived from VCUSM2; has 1 copy of Δ*ctx* operon; 1 copy of Δ*ctx* operon::ΔaphA; Δ*hem*A	Universiti Sains Malaysia
VCUSM20	*V. cholerae* O139 ALA auxotroph strain that was constructed from VCUSM14. It has 1 copy of Δ*ctx* operon, 1 copy of Δ*ctx* operon::Δ*aph*A, *rtx*C::*aph*A and Δ*hem*A genes.	This study
VCUSM21	*V. cholerae* O139 ALA auxotroph strain that was constructed from VCUSM20. It has 1 copy of Δ*ctx* operon, 1 copy of Δ*ctx* operon::Δ*aph*A, Δ*rtx*C/A and Δ*hem*A genes.	This study
VCUSM21P	*V. cholerae* O139 ALA non-auxotroph strain that was constructed from VCUSM21. It has 1 copy of Δ*ctx* operon, 1 copy of Δ*ctx* operon::Δ*aph*A, Δ*rtx*C/A and *hem*A genes.	This study
Plasmids		
pWM91-*rtx*C::*aph*A	pWM91 with *rtx*C::*aph*A fragment	USM
pWM91-Δ*rtx*C/A	pWM91 with Δ*rtx*C/A fragment	USM
pCVD-*hem*A/M	pCVD442 with *hem*A-*hem*M fragment	USM

### Animal

New Zealand White adult rabbits and BALB/c mice which were bred in the Animal House, University Sains Malaysia (USM) Health Campus, were used in this study. New Zealand White adult male rabbits (weighing 2.0–2.5 kg at the onset of the experiments) were used only if they were healthy and showed no evidence of diarrhoeal disease during the time period. All procedures involving the use of animals had the approval of the Animal Ethics Committee (AEC), USM. The institution's law on the care and use of laboratory animals was followed.

### Construction of auxotropic vaccine strains: VCUSM20 and VCUSM21

To construct *rtx*C mutation in VCUSM14, VCUSM14 was conjugated with *E. coli* BW 20767 *λpir* carrying pwm91 *rtx*C::*aph*A. The conjugation was done at 37°C incubation on LB agar supplemented with ALA. After 5 hours, bacteria were transferred from the plate to LB broth and suspended. The cell suspension was diluted, and then spread on LB agar supplemented with ALA and consisting of Kan/Amp/Poly, which was used to obtain co-integrate/transconjugants and to non-counter-select the *E. coli* BW 20767 *λpir* donor strain and VCUSM14. The plates were incubated overnight at 37°C for 12–16 hours. Colonies/transconjugants from the plates were selected and confirmed by antibiotic resistance and also PCR. The confirmed transconjugant was suspended in NaCl free LB broth with 10% sucrose and with 80 µg/ml ALA added and incubated at 21–25°C for 12–16 hours. The suspension was plated at various dilutions on NaCl free LB agar with Kan, , supplemented with ALA and solidified with sucrose (10%, w/v) and incubated at 21–25°C. Selection of the desired clone was based on its ability to be Suc^R^/Kan^R^/Amp^S^/Poly^R^, inability to grow without ALA supplement and ability to produce yellow colonies on Thiosulfate-Citrate-Bile-Sucrose Agar (TCBS) supplemented with ALA. The constructed strain was designated as VCUSM20 and and the rtxC deletion was further confirmed by performing PCR using primers *rtx*C-R1 (5′-GGTGGCCAGTGGAATCTTCCA-3′) and *rtx*C-F1 (5′-AAAGTCGGTATTGCGGCACGC-3′). The above procedure was followed for the construction of Δ*rtx*C/A mutant but with modification. *E. coli* BW 20767 *λpir* carrying pwm91 Δ*rtx*C/A was conjugated with VCUSM20. Clone counter-selection and elimination of the *E. coli* donor strain and VCUSM20 were performed by plating on LB Amp/Poly agar plate without adding Kan. Desired clone selection was based on its ability to be Suc^R^/Kan^S^/Amp^S^/Poly^R^, not to grow without ALA supplement and produce yellow colonies on TCBS agar supplemented with ALA. The obtained strain was named as VCUSM21. This strain construct was was screened with the similar primers as mentioned above, *rtx*C-R1 and *rtx*C-F1, for confirmation. List of plasmids that were used in the present study is given in Table 2.

**Table 2:** Primers used in this study with their sequences, annealing temperature and functions.

Primer	Sequence 5′ — 3′	T_A_ °C	Function
SacBF2	ATGAACATCAAAAAGTTTGCAAAA	58	Amplification of *sac*B gene in merodiploids and to confirm the elimination of suicidal plasmid backbone in *V. cholerae*
SacBR	TTATTTGTTAACTGTTAATTGTCC	58	Amplification of *sac*B gene
KanFse-2F	AGCGGCCGGCCGCTTACATGGCGATAGCTA	56	Amplification of *aph*A cassette in *V. cholerae*
KanFse-R	ATAGGCCGGCCTCAGAAGAACTCGTCAAGA	60	Amplification of *aph*A
RtxC F	TTATCAGAGATGGCAGCACC	66	Amplification of *rtx*, Δ*rtx*C/A and *rtx*::*aph*A genes in *V. cholerae*
RtxC R	CTTGTCCACCGCTGTAGCCT	66	Amplification of *rtx*, Δ*rtx*C/A and *rtx*::*aph*A genes in *V. cholerae*
VHAF	ATGTCTTTGCTTGCCATTGG	56	Amplification of *hem*A and Δ*hem*A genes in *V. cholerae*
VHAR2	GTTCAGATCGTCAAGACCTA	56	Amplification of *hem*A and Δ*hem*A

T_A_ annealing temperature.

### Construction of non-auxotropic vaccine strains: VCUSM21P

In order to construct a vaccine candidate from VCUSM21 that could synthesize its own ALA, the WT *hem*A gene was deleted and replaced with the Δ*hem*A gene. Construction was performed as mentioned above with modification. For VCUSM21P construction, *E. coli* BW 20767 *λpir* carrying pCVD *hem*A/M was used as donor strain and VCUSM21 were used as recipient strains. Bacterial conjugation was done on LB agar supplemented with ALA and merodiploids were obtained from LB agar with Amp/Poly but without ALA supplement, allowing selection for clones that could synthesize their own ALA. The recombinant strains were derived directly by growing the merodiploid in LB broth lacking NaCl with 10% sucrose but without ALA and plated as mentioned above at various dilutions on LB agar, without NaCl, and solidified with sucrose (10%, w/v), but without ALA supplement. Through this selection process, cells which still remain as *hem*A mutant were not able to grow in the broth medium and on the plates, thus not selected. The obtained clones were further confirmed by testing their ability to be Suc^R^/Kan^S^/Amp^S^/Poly^R^, ability to grow without ALA supplement and ability to produce yellow colonies on TCBS agar. The construction was confirmed successful only if the clone could synthesize its own ALA, which is necessary for *hem* biosynthesis. Confirmation was done by testing the clone ability to grow on LB agar without ALA. The ALA producing clone was designated as VCUSM21P. Colonies were serologically confirmed as *V. cholerae* O139.

### GM1 enzyme-linked immunosorbent assay

Enzyme-linked immunosorbent assay analysis was performed to determine the inactivated CT, CT B subunit and CT A subunit in VCUSM21P. Briefly, ELISA plates (MaxiSorp; Nunc) were coated with 100 ng/well of commercially available type lll GM1 ganglioside in 60 mM carbonate buffer (pH 9.6) at 4°C. Commercially available cholera toxin was used as a standard with the starting concentration of 100 ng followed by 1∶2 dilutions. Culture was prepared by incubating 1×10^6^ colony forming unit (CFU) of Wild Type (WT) or VCUSM21P cells in 10 ml LB broth for 18 hours. 100 µl of each culture supernatant was added into the wells. As primary antibody, rabbit anti-cholera toxin polyclonal antibodies, rabbit polyclonal anti-CTB antibody and rabbit polyclonal anti-CTA antibody (1∶2000 diluted in PBS), were used. Whereas, anti-rabbit IgG antibodies conjugated with HRP (1∶3000 diluted in PBS) were used as secondary antibodies. To complete the reaction, the wells were added with 2,2′-azino-bis(3-ethylbenzthiazoline-6-sulphonic acid) (ABTS). Reaction was allowed to take place for 30 minutes in the dark and stopped before readings were taken using Multiskan EX microtiter plate reader at 405 nm with reference to 495 nm.

### Cytotoxicity Assays

HEp-2 cells were seeded into 24-well tissue culture plate at 1×10^5^ cells per well in RPMI medium 1640 with 10 mM HEPES buffer, 2 mM glutamine, 10% fetal calf serum and supplements (without antibiotics) as described by Lin *et al.* [16]. A subconfluent monolayer of HEp-2 cells in a 24-well tissue culture plate was infected with VCUSM21P or WT at a multiplicity of infection (MOI) of 100. PBS was added to the monolayer of HEp-2 cells as a control. The plate was incubated in 5% CO_2_ at 37°C for 1 h before viewing live cells under an inverted microscope. Cytotoxic activity was identified if cell rounding of live cells became visible.

### Mouse Colonization Assay

Eight 3–5-days-old BALB/c mice were divided into four groups and kept fasting by separating them from their mothers 1 hour prior to performing the mouse colonization assay as described before by Angelichio *et al.* [34]. Mice from the first group were inoculated with VCUSM21P strain, the second and third were inoculated with VCUSM20 and VCUSM21 respectively, and the fourth group was inoculated with WT. The inoculation was performed intragastrically, whereby 1×10^6^ cells of strain in 50 µl ice-cold NS was fed to the mice by 21 G gavage tube inserted 1 cm deep into the oesophagus. After 16–18 h, all mice from each group were euthanized using chloroform, and their gut were excised before placing into 15 ml centrifuge tube containing 5 ml of sterile LB-20% v/v glycerol. They were then transferred into sterile potter homogenizer and homogenized in LB-20% v/v glycerol. The presence of viable vibrios from the mice intestine were assessed by serially diluting the homogenates and plating 100 µl of each diluted suspension onto LB agar containing Poly (for WT and non-autotrophic mutant) or LB agar containing Poly, supplemented with ALA (for autotrophic mutants). The plates were incubated for 16 h at 37°C. The numbers of viable tested strains colonizing the intestine were quantified by counting subsequent colonies after 16 hours of incubation.

### Immunization of rabbits

Four New Zealand White rabbits were used for vaccine administration as performed by Thungapathra *et al.* [35] with modifications. Prior to vaccination, the rabbits were orally administered twice (with an interval of 2 days) with 125 mg/kg of Metronidazole and 164 mg/kg of Sulfaquinoxaline. The rabbits were fasted 24–36 hours before the first vaccination but water was given ad libitum. They were anaesthetized with an intramuscular injection of ketamine (35 mg/kg), Xylazine (4 mg/kg) and Acepromazine (1 mg/kg). Before vaccinating the rabbits with VCUSM21P, they were intravenously administrated with Cimetidine (50 mg/kg) to inhibit HCl secretion and followed by NaHCO_3_ administration to neutralize their gastric pH. Rabbits were given a first vaccination with 1×10^10^ CFU of VCUSM21P in 10 ml LB broth, on day 0. To inhibit the peristaltic movement, 1 ml of morphine (10 mg) was injected intra-peritoneally before they were returned to their cages. The second vaccination was administered to the rabbits on day 14.

### Assessment of vibrios shedding after immunization

To assess the excretion of vibrios or shedding of challenge vibrios in coproculture, briefly, rectal swab was performed daily after first and second vaccination, up to 5 days. The rectal swab was incubated at 37°C for 5–6 h in alkaline peptone water [APW; peptone 1% (wt/vol), NaCl 1% (wt/vol)]. 100 µl of the enriched culture was then streaked on TCBS and incubated at 37°C for 16 hours. Any appearance of thin-edged yellow colonies on TCBS agar was recorded.

### Serology

The pre-immune sera samples were collected from the ear vein of the rabbits before immunization. The post-immune sera collections were done at an interval of 7 days, up to 4 weeks. All collected serum samples were stored at −20°C until tested for the presence of vibriocidal antibodies, as well as for the specific CT and O139 LPS antibodies.

### Antibody assays

CT and O139 LPS -specific antibody titres in serum were determined by performing ELISA. The ELISA plates were coated with either commercially available CT or O139 LPS extracted in the laboratory for anti-CTAB IgG or IgA, and anti-LPS IgG or IgA detection, respectively. Briefly, 96-well microtiter plates (Maxisorp; Nunc) were coated overnight at 4°C with 50 ng/well of either CT or 50 ng/well of O139 LPS in carbonate buffer (pH 9.6). Serum samples were diluted to 1∶10–1∶1280 in PBS and used as the primary antibody. The diluted sera were suspended and incubated in the presence of anti-rabbit IgG-HRP (100 µl; 1∶5000 in PBS) as the secondary antibody at 37°C for 30 min or IgA-HRP (100 µl; 1∶3000 in PBS) at 37°C for 1 h. To complete the assay, 100 µl of ABTS (1 mg/ml) was added into the wells and subsequently incubated at 37°C in the dark for 30 min. Plates were read at 405 nm with reference to 495 nm using a Multiskan EX microtiter plate reader. The antibody titer is defined as the highest dilution that gave an optical density (OD) greater than that of the preimmune serum.

### Vibriocidal antibody assay

Sera from rabbits immunized with VCUSM21P were assayed for vibriocidal antibodies against CIR134 strains of *V. cholerae* following an assay protocol as described previously by Attridge et al. with modification [36]. Serum was inactivated at 56°C for 30 min. In the wells of 96-well microtiter plates, the inactivated serum was serially diluted in PBS (1∶10 to 1∶20480). 50 µl of a chilled suspension comprising the indicator bacteria (CIR134 in a final concentration of 1×10^5^ cells/ml) and fresh commercially available normal rabbit serum (20% v/v) as a source of complement factor was then added into the wells. After incubation at 37°C for 60 min, the suspensions from the microtiter plates were plated on LB/polymyxin plates and incubated at 37°C for 16 h. Vibriocidal titer was defined as the highest serum dilution causing 100% bacterial killing as compared to the pre-immune sera.

### Protective efficacy in RITARD

RITARD (Reversible Intestinal Tie Adult Rabbit Diarrhoea) assay was performed on the non-immunized and immunized rabbits by the method of Rusell et al. [37]. In RITARD model, the caecum was ligated as close as possible to the ileocaecal junction following application of a reversible knot tie at the ileum, approximately 10 cm away from the ileocaecal junction. Rabbits were challenged with 1×10^9^ CFU WT which was injected into the jejunum 10 cm distal to the stomach. Control group of animals was included. The knot was removed 2 hours after administration of WT. All the rabbits undergoing RITARD experiment were observed every 6 h for clinical signs of infection such as diarrhoea symptoms or death, up to 5 days. Shedding of vibrios in faeces was monitored. The animals were euthanized with an intravenous injection of sodium pentabarbitone on day 6 and microbiological testing was performed by collecting mucus or fluid from the intestine and plating on TCBS.

### Reactogenicity studies in ligated ileal loop model

The surgical procedures employed in this study have been described previously by Thungapathra et al. with modifications [35]. The rabbits were kept fasting for 24 hours prior to the surgery. Anesthesia was induced as described in immunization. Following anesthesia, an incision was made along the linea alba of the abdominal wall. The ileum was exposed and ligation was performed approximately 10 cm away from ileocaecal junction. Five cm long loops were ligated on the small intestine, leaving 1 cm loops between them. 1 ml sterile NS was injected into the lumen of the control loops. Others were inoculated with 1 ml of a suspension of VCUSM21P or WT strains in NS containing approximately 1×10^6^ and 1×10^8^ CFU/ml. After 18 h, rabbits were euthanized to assess the reactogenicity. Reactogenicity was described in fluid accumulation ratio, FA, the ratio of fluid in the intestinal loop (milliliters) to length of the loop (centimeters).

## Results

### Construction of vaccine strains

**Figure 1:**
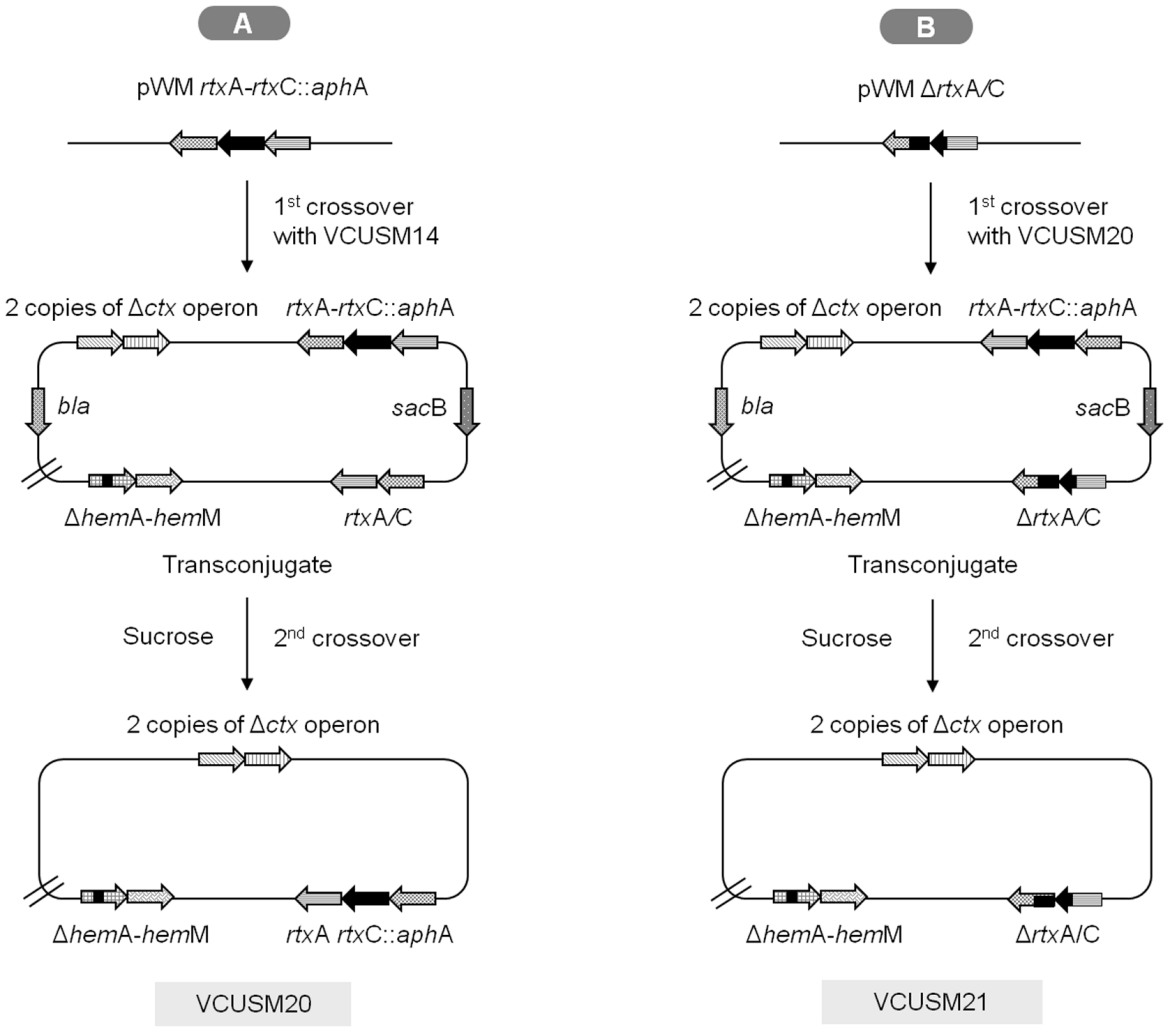
Diagram of the auxotrophic strains, VCUSM20 and VCUSM21, construction. The diagram is a schematic representation of mutation introduced at the *rtx* gene cluster on VCUSM14 (A) and VCUSM20 (B) chromosomes. It shows the double copies of Δ*ctx* operon, Δ*hem*A-*hem*M genes and *rtx*C::*aphA* genes in VCUSM20, and *rtx*C::*aph*A replaced with Δ*rtx*C/A in VCUSM21. Gene designations: Δ*ctx*A operon, mutation in CT A subunit with complete deletion of accessory cholera enterotoxin (*ace*) and zonula occludens toxin (*zot*); repeat-in-toxin (*rtx*); *bla*, β-lactamase; *sac*B, *Bacillus subtilis* counterselectable marker *sacB* which encodes levan sucrase.

**Figure 2:**
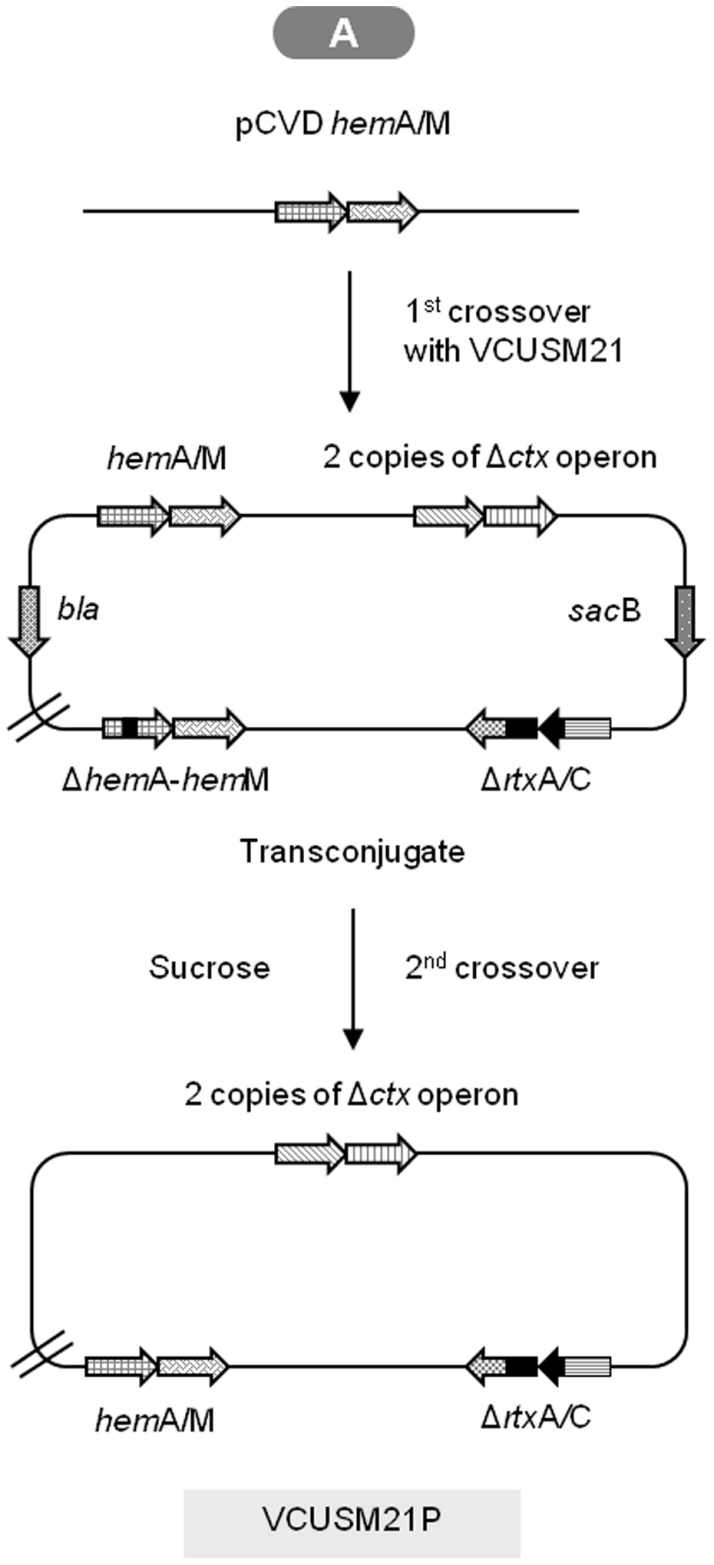
Diagram of the prototrophic vaccine candidate, VCUSM21P, construction. The diagram is a schematic representation of Δ*hem*A mutation removed from VCUSM21 chromosome, and replacing it with the native *hem*A gene from *V. cholerae* O139. It shows the double copies of Δ*ctx* operon, Δ*rtx*C/A, and *hem*A/M genes in VCUSM21P. Gene designations: as Figure 1.

The vaccine candidate VCUSM20 was successfully obtained by introducing the *rtx*C::*aph*A gene into the chromosome of VCUSM14 via suicide plasmid integration into the chromosome and subsequent sucrose counterselection (Figure 1). Using a similar technique, VCUSM21 was constructed by introducing Δ*rtx*C/A gene into the chromosome of VCUSM20 and replacing the *rtx*C::*aph*A gene (Figure 1). The mutants were successfully confirmed not to carry the native *rtx*C/A gene by PCR. Finally, the construction was completed by removing the mutated *hem*A gene and replacing it with *hem*A gene of the WT that could code the functional glutamyl-tRNA reductase (Figure 2). The selected clone was confirmed to be able to grow on all test plates and produced the expected bands after PCR amplification. The mutant is a Suc^R^/Kan^S^/Amp^S^/Poly^R^ strain and it is able to produce yellow colonies on TCBS agar. The new vaccine candidate, VCUSM21P, is a *ctx*A, *ace*, *zot* and *rtx*C/A mutant that has lost its parent auxotropic characteristic, its dependency on ALA supplement for survival.

### Glutamyl-tRNA reductase production and ALA test

Production of glutamyl-tRNA reductase is required for ALA biosynthesis, and consequently for *hem* biosynthesis in VCUSM14. It has limited generation without ALA supplement due to its inability to encode glutamyl-tRNA reductase. In order to determine whether VCUSM21P has similar function in encoding glutamyl-tRNA reductase as the WT, VCUSM21P and the WT strains were grown in LB broth and on LB agar plate without ALA supplement. VCUSM20 and VCUSM21 strains were also included in this test. Both VCUSM20 and VCUSM21 had growth impairment in LB broth, and neither of them survived on LB agar without ALA supplement. On the contrary, VCUSM21P and the WT strain were surviving and growing in LB broth/on agar without ALA supplement, confirming VCUSM21P independency on ALA supplement for its survival or growth. Replacement of the mutated *hem*A gene with its homologous WT gene resulted in functional glutamyl-tRNA reductase encoded by VCUSM21P, as the WT cells could, and ultimately lead to the synthesis of *hem* within its cells.

### Cytotoxicity assay

**Figure 3:**
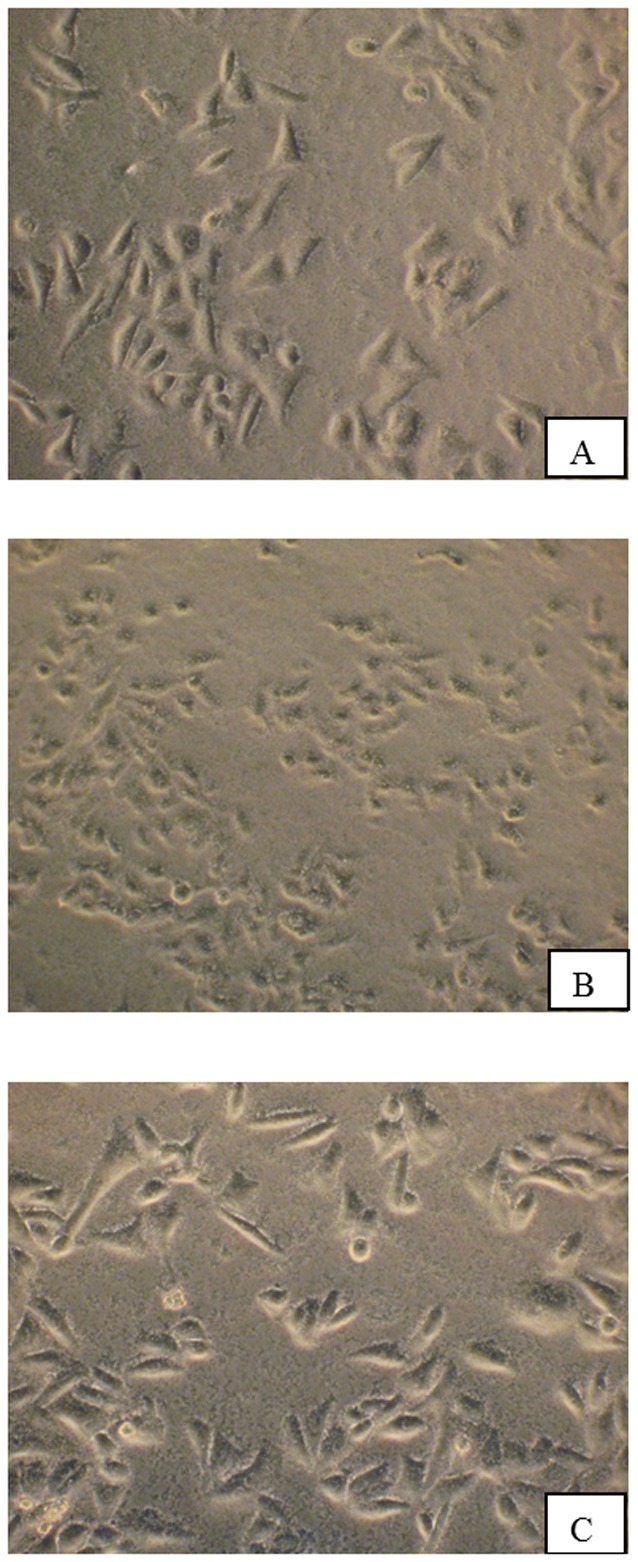
Inverted microscopy reveals morphological changes on HEp-2 cells after infection with WT (B) and VCUSM21P (C), at a magnification of ×40. As control, HEp-2 cells in PBS (A) were included in the cytotoxic assay. HEp-2 cells were incubated for 1 hour with WT and VCUSM21P cells at multiplicity of infection (MOI) of 100. B shows rounded HEp-2 cells indicating cytotoxic effects. A and C show HEp-2 cells with normal morphology indicating absence of cytotoxicity.

To evaluate the cytotoxicity of the constructed vaccine candidate, HEp-2 cells were infected with VCUSM21P and WT infected cells were included for comparison. Cytotoxic effect is appreciated/recognized as rounding up of infected cells due to depolymerization of cytoskeleton actin. Because the cytotoxic activity can be easily monitored by microscopic analysis of HEp-2 cell line, the ability of the vaccine candidate to depolymerize actin was discerned by observing the infected HEp-2 cells under an inverted microscope. VCUSM21P infected cells failed to demonstrate cell rounding activity as shown in Figure 3C, indicating absence of cytopathic effects on HEp-2 cells. By contrast, WT exhibited massive cell rounding of infected HEp-2 cells indicating its cytotoxic effect on them (Figure 3B). The absence of cell rounding, compared with the cytotoxic activity created by the WT on the HEp-2 cells, suggests that VCUSM21P has lost the fundamental role in the actin depolymerization.

### Non-toxic CT production

**Figure 4:**
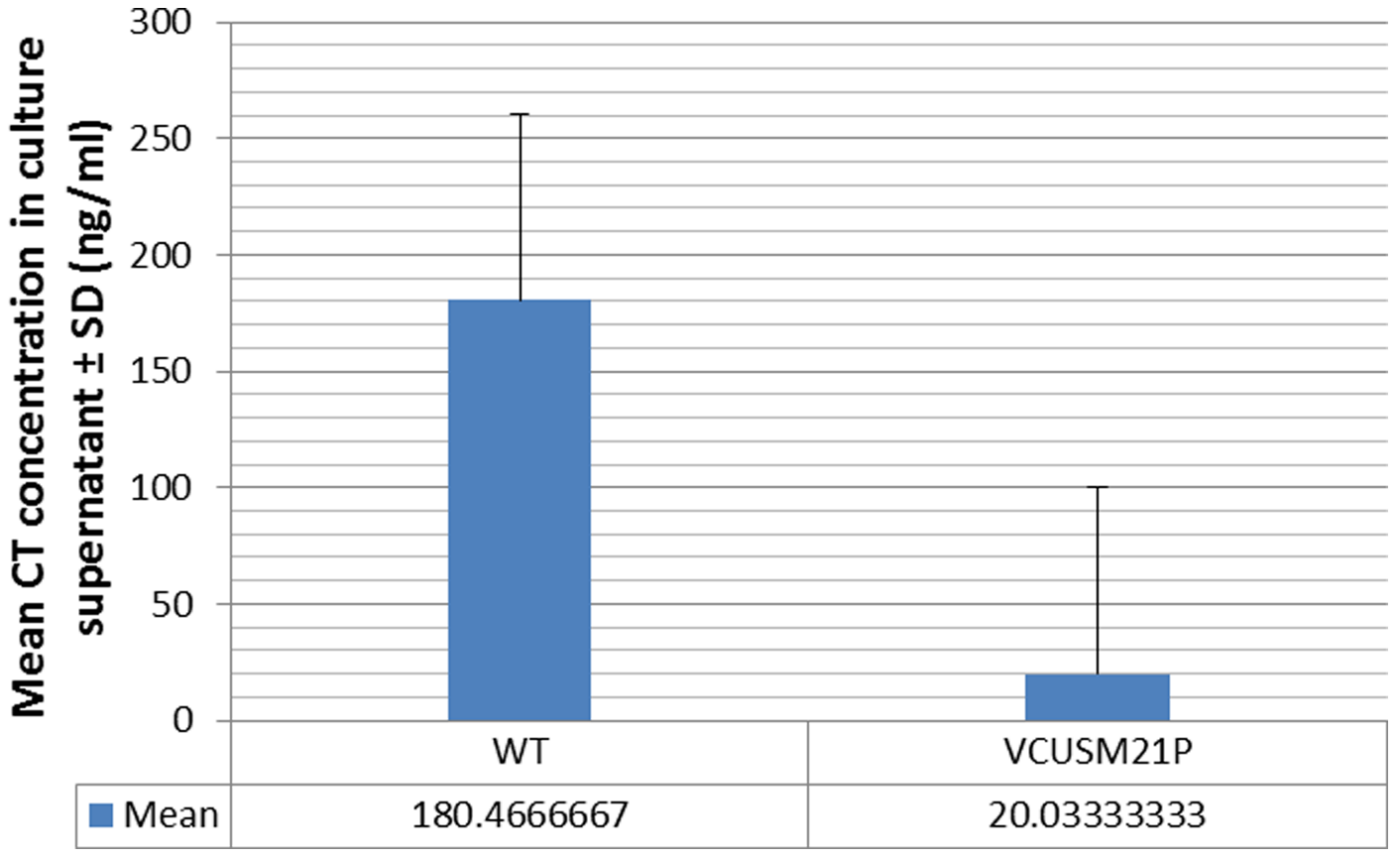
Cholera toxin production by vaccine candidate VCUSM21P and WT strain. Values are expressed as mean± standard deviation.

The production of immunogenic CT which consists of five identical B subunits and a non-enzymatically active A subunit by VCUSM21P was measured by GM1 ganglioside-binding ELISA.Detectable amounts of CT were present in the culture broth of WT, as well as VCUSM21P, as shown in Figure 4 & Table 4. WT strain produce native CT, but the vaccine strain produces inactive/mutant CT. The *ace*, *zot*, *ctx*A and *rtx*C/A mutant was confirmed to encode the non-toxin CT successfully, but the production was more diminished compared with WT. The rationale for the diminished amount is not known. It is possible that several genomic alterations that VCUSM21P has undergone during its creation through the homologous recombination or sucrose counter-selection could be the explanation for the reduction in CT gene expression and CT production. Alternatively, the explanation may well be due to the mutation of *ctx*A found at the region of *ctx* operon.

**Table 4:** Cholera toxin, CT A and CT B production by vaccine candidate VCUSM21P and WT strain.

Strain	Mean concentration in culture supernatant (ng/ml)		
	CT A	CT B	CTAB
WT	146.26	174.40	180.47
VCUSM21P	18.12	141.18	20.03

### Colonization

The colonization ability of VCUSM20, VCUSM21 and VCUSM21P in the infant mice intestine was assayed to test the capability of these strains to attach to the eukaryotic cell surface of the host prior to induction of an immune response. Interpretation of the data obtained, expressed in terms of average CFU recovered after inoculating 1×10^6^ CFU, from infant mice intestine showed that colonization of both VCUSM20 and VCUSM21 did not take place in the infant mice bowel. Nevertheless, VCUSM21P colonized exceptionally well, an average of 1.55×10^6^ CFU of VCUSM21P was recovered from the infant mice intestine. However, the average number of recovered cells of this vaccine candidate was found to be less when compared to viable cells recovered from mice inoculated with the WT. The ability of VCUSM21P to encode for glutamyl-tRNA reductase, and thus to synthesize ALA, has dramatically improved the colonization ability of it. We concluded that the presence of *hem*A mutation in its parent vaccine caused a remarkable effect on the intestinal colonization of *V. cholerae* in mice. VCUSM21P is a good colonizer of the small intestine and preferred vaccine candidate for vaccination against cholera due to its potential means to stimulate effective immune response compared with its respective parent strains.

### Reactogenicity study

**Figure 5:**
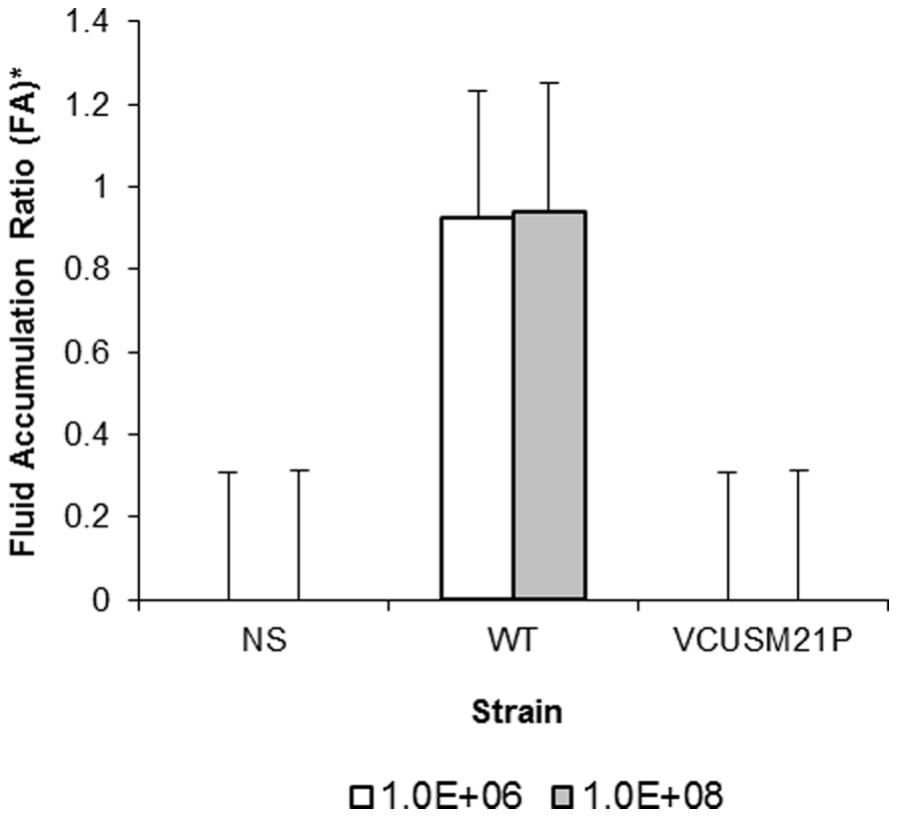
Fluid accumulation ratio in loops of unvaccinated rabbits.

In order to assess whether this newly constructed vaccine is causing reactogenicity, we analyzed the fluid accumulation ratio (the mean fluid (ml)/length (cm) ratio of loop) of the WT in parallel with VCUSM21P by ileal loop assay. As indicated in Figure 5, there was an increase in the fluid accumulation ratio in loops injected with 1×10^6^ and 1×10^8^ CFU of WT. These characteristics would appear to correlate with the symptoms of acute cholera, i.e. having frequent loose or liquid bowel movements. Differing to that anticipated for the WT, VCUSM21P did not show any sign of hemorrhage or reactogenicity, FA ratio in both 1×10^6^ and 1×10^8^ CFU of VCUSM21P administrated loops were ≥0 (Figure 5). Inability of VCUSM21P to induce any fluid accumulation or potentially diarrhoea could be principally due to its non-functional CTA that could not influence the intracellular cAMP activation. Moreover, the positive effects on fluid accumulation in intestinal loops could be greatly attributed to the absence of *ace*, *zot*, *rtx*C/A and *ctx*A toxin activity as a whole.

### Antibody responses in serum following oral immunization

#### Antibodies against CTAB and LPS O139

As can be seen in Table 3, all the immunized rabbits were found to have antibodies against CTAB and LPS O139 in their sera. Seven days after the first immunization, a low geometric mean titer (GMT) of anti-CTAB IgG/IgA and anti-LPS IgG/IgA were detected in all sera of immunized rabbits, as compared to the basal titer. This proves the very early immune responses induced by the administered vaccine. GMT records of the following weeks showed that all the immunized rabbits kept producing rising antibodies of O139 LPS IgG/IgA and anti-CTAB IgG/IgA. The majority of immunized rabbits were found to acquire the highest level of protection against CT or O139 LPS on week 3 or 4, as evidenced from their serum anti-O139 LPS IgA titer having their peaks at week 3, and anti-O139 LPS IgG and anti-CTAB IgG/IgA titers having at week 4. The geometric mean fold increase from baseline of O139 LPS IgG induced by VCUSM21P on week 1 was statistically significantly (*p*≤0.05). Immunological study has confirmed that VCUSM21P is capable of inducing humoral immune responses against CT and O139.

**Table 3:** Antibodies titers of rabbits immunized with VCUSM21P.

Vaccine candidateá	Antibody	GMT Pre-immune	Week	GMT (SD) ğ ^:^ Post-immune	*p*-Value
VCUSM21P	CTBA IgG	10	1	11.89 (5)	0.317
			2	23.78 (32.02)	0.102
			3	95.14 (60)	0.066
			4	113.14 (46.19)	0.063
	CTBA IgA	10	1	14.14 (5.77)	0.157
			2	33.64 (10)	0.059
			3	47.57 (20)	0.059
			4	135.62 (40)	0.059
	O139 LPS IgG	10	1	40 (0)	0.046*
			2	226.27 (0)	0.059
			3	755.08 (240)	0.059
			4	883.80 (300)	0.059
	O139 LPS IgA	10	1	28.28 (11.55)	0.063
			2	47.57 (20)	0.059
			3	95.14 (40)	0.059
			4	67.27 (20)	0.059
	Vibriocidal	10	1	320 (0)	0.046*
			2	538.17 (452.55)	0.066
			3	1810.19 (739.01)	0.063
			4	2560 (0)	0.046*

á Immunization with 1×10^10^ CFU of vaccine candidates.

ğ Geometric Mean Titer (GMT) antibody in sera of four animals.

Significant difference between the pre-immunization and post-immunization levels is shown by * (*p*≤0.05, Wilcoxon signed rank test).

GMT is defined as “the ‘n’th root product of ‘n’ numbers”, (a_1_a_2_…a_n_)^1/n^.

#### Vibriocidal antibodies

Furthermore, VCUSM21P was found to stimulate an effective antibacterial antibody response capable of killing vibrios in all rabbits as the detected vibriocidal antibodies were higher in all post-immune sera than that of the pre-immune sera (Table 3). An elevated vibriocidal antibody titer, 320 GMT, was recorded in the first week, indicating the level of protection provided by VCUSM21P against cholera at the very early stage following immunization. At week 2, VCUSM21P elicited a ∼538-fold increase in vibriocidal antibody titer compared with that of the basal titer, 10 GMT. The antibodies continued to rise to 1810 GMT in the following week. The highest level of vibriocidal antibody, 2560 GMT, was detected at week 4. All immunized rabbits were confirmed to develop the preferred protection against cholera after vaccination using VCUSM21P.

### Bacterial shedding following the oral vaccination

Shedding of vibrios after vaccination, an indication of the intestinal colonization and multiplication of the vaccine candidate in the intestine, by the rabbits was assessed from their rectal swab, which were enriched in APW and later grown on TCBS agar plate. Among the 4 rabbits, two of them did not shed vibrios after the first immunization, but shedding by the other two rabbits lasted for 1–2 days, representing intestinal passage. After the second immunization, shedding of vibrios was not found in any of the rabbits.

### Protection provided by immunization/Protection obtained from immunization

#### Challenge study using RITARD model

In the RITARD experiment, the immunized animals were challenged with freshly cultured 1×10^9^ CFU of WT strain in sterile saline via intradoudenal injection, to assess the immunity induced by VCUSM21P. The immunized-animals which were examined twice daily up to 5 days, had a positive clinical outcome, without any symptoms of diarrhoea or mortality. Microbiological examination for the presence of WT colonies on TCBS agar plate revealed that after five days, WT strains were not found in the intestine of immunized rabbits. As reported previously [38] and found in this study, 100% of the non-immunized rabbits had a fatal clinical outcome following infection with WT at a dose of 1×10^9^ CFU, where they experienced severe diarrhoea and eventually did not survive.

#### Reactogenicity in ileal loop assay

An *in vivo* ileal loop assay, as described above, was used to estimate the protection that VCUSM21P could provide against WT. To determine the capacity of cholera toxin and also other toxins to induce reactogenicity in vaccinated rabbits, the presence of fluid was assessed in the intestinal loops that were challenged with different CFUs of WT. 3 weeks after second immunization, 1×10^2^–1×10^8^ CFU of WT were injected into the loops. As mentioned above, in non-immunized rabbits, 1×10^6^ and 1×10^8^ CFU of WT infection caused extensive fluid accumulation in the intestinal loops. However, in immunized rabbits, only the loops challenged with 1×10^8^ CFU WT had fluid present, but not in loops challenged with 1×10^6^ CFU. This signifies a reduction of the elevated cAMP level in the intestinal mucosal cells following WT challenge in immunized rabbits. In others, loops receiving 1×10^2^ to 1×10^5^ and 1×10^7^ CFU of WT, were also found to be protected; they had no fluid accumulation.

## Discussion

Cholera is the vaccine-preventable killer of adult and children in the developing world. Understanding of *V. cholerae* virulence factors has increased greatly during recent years but remains incomplete. The mechanisms that regulate reactogenicity of *V. cholerae* are remarkably conserved within the *ctx* operon of the phage genome that resides on its chromosome. However, few studies have shown that the other virulent proteins might be contributing to, or are regulating, residual reactogenicity in the host. The isolation of *V. cholerae* WO7, a O1 serovar, from patients with diarrhoea indicated that virulent *ctx*A, *zot*, and *ace* genes were not the only genes involved in gastrointestinal disease as WO7 is devoid of all these genes of phage belongings [39]. Previous studies also have shown that vaccinees were experiencing mild diarrhoea after vaccination with live O1 *V. cholerae* vaccine candidate that were *ctx*, *zot* and *ace* mutant [32].

Besides toxins produced by the genes belonging to the CTXΦ lysogen, the repeat-in-toxin (RTX) exoprotein could have a significant role in inducing diarrhoea in host infected with lethal vibrios. RTX toxin was suggested to contribute to the emergence of pathogenic non-O1/non-O139 strains [40]. Many RTX mutants of microbial species have been described that exhibit a defect in or loss of cytotoxic activity [16], [41], [42]. This is due to the inability of the defective RTX protein to bind actin and catalyze the cross-linking reaction that leads actin monomers into dimers, trimers, and higher multimers. According to Lin *et al*., the cytotoxic activity is not caused by *ctx*AB, *zot*, or *ace* genes, but only by the *rtx* gene cluster [16]. Moreover, RTX toxin produced by *V. cholerae* O1 El Tor was demonstrated to contribute to the severity of acute inflammatory responses [17] associated with interleukin (IL)-6 and murine macrophage inflammatory protein (MIP)-2, suggesting that the toxin induces cellular events that promote inflammation. This toxin also promotes the production of proinflammatory substances such as IL-1, TNF, nitric oxide, and eicosanoids [43]. The above description and suggestions link RTX toxin to gastrointestinal inflammation.

As safety is one of the main concerns for attenuated live vaccines, in this work, we extended the safety studies of the VCUSM14 vaccine candidate against *V. cholerae* O139. This was performed by constructing a new vaccine candidate by disrupting the *rtx*C/A gene on chromosome of VCUSM14. To confirm that RTX toxin lost its activity due to mutation, we analyzed the ability of VCUSM21P to produce cell rounding of HEp-2 cell line. Mutation of both the *rtx*A and *rtx*C genes completely abolished the cell rounding activity of O139 strain [40]. Deletion of a chromosomally encoded *rtx*C/A in VCUSM21P led to a reduction or absence of cytoxicity to the HEp-2 cells; it exhibited defective cell rounding activity. This gained insight suggests that VCUSM21P has lost the depolymerization of actin cytoskeleton. Based on this observation, it is suggested that this new vaccine could play a role in intervening the cytotoxin mechanism, which may possibly be associated with factors contributing to the development of mild diarrhoea.

In previous studies, we have designed attenuated VCUSM2 and VCUSM14 vaccine candidates; both attenuated vaccines are metabolic auxotroph recombinant which requires exogenous supplement of ALA for survival. Unlike its parent strain VCUSM2, which has impairment in encoding for glutamyl-tRNA reductase, VCUSM14 has additional mutations at the *ctx* operon, where the double copies of *ace* and *zot* genes were removed and its *ctx*A, in dual copies, has been rendered non-virulent.

In VCUSM14, ALA supplement is required for the hem synthesis and it is unable to grow without ALA; hemA deficiency obviously affected the cell growth as hem is essential for many cellular processes. In this study, due to inability of VCUSM14 to encode ALA *in vivo*, the vaccine candidates derived from it were found to have reduced colonization ability in the infant mouse intestine. A reduction in the number of colonizing cells was also reported in our previous studies, where the auxotrophic VCUSM2 did not colonize as the WT [38]. For the induction of effective immune responses, successful and efficient infection mechanism is required. As colonization, a process of breaching mucosal barriers by the live vibrios and their attachment to receptors of epithelial cells, is required to initiate infection, improvement in colonization capacity of VCUSM14 prototype was essential.

Therefore, in this study, besides mutating the *rtx*C/A by allele replacement technique, the probable inability of VCUSM14 to induce high and sustained titers of antibodies against cholera was addressed. The WT *hem*A gene was introduced in VCUSM21P. Analysis of mice colonization assay revealed a significant increase in colonization ability of VCUSM21P compared to that of VCUSM21. Given that the VCUSM21 did not survive after overnight incubation without ALA supplement on LB agar, and correlating this with the mouse colonization assay, it was concluded that due to inability to synthesize ALA, VCUSM21 cells could not survive without ALA supplement in the intestine whereas the VCUSM21P could survive VCUSM21 could still mimic the disease development mechanism of cholera, but not as fully as the WT. More proficient disease development mechanisms could be expected to be established by the VCUSM21P vaccine candidate, referring to its prolonged survival capacity. VCUSM21P live attenuated vaccine strain was only recoverable in the stool of 2 of 4 rabbits for 1–2 days after a large large oral inoculum with the first inoculation and not at all in any of the 4 rabbits after their second inoculation. However, it is anticipated that attenuated strain will colonize the intestinal mucosal surface for at least a few days in order to present many of the antigens that may be associated with protection. The mice data are uninterruptable from the level of colonization since the mice were sacrificed 16–18 hours after oral inoculation and therefore, intestinal detection would not be consistent with colonization vs. transient intestinal passage.

All four rabbits given the vaccination regime displayed no sign of any residual diarrhoea, and these animals had developed effective immunity against *V. cholerae* O139, evident from the high level of antibodies against CTAB and LPS O139. Overall, they also had the best known immunological correlation of protection against cholera, the vibriocidal antibody, at elevated level. The dose of vaccine used in this experiment has been rather similar to that administrated previously [38] and, in the majority of animals tested, induced substantial protective antibodies. Two immunization regimens were able to greatly reduce WT bacterial replication in the main site of bacterial burden, the mucus of the intestine. Consistent with the high vibriocidal antibody titers, all animals which had received the vaccine demonstrated no cholera symptom after the WT challenge.

*V. cholerae* O139 is no longer exiting, but outbreak could happen in future. Despite best efforts, a fully effective vaccine is still to be obtained [44]. It is important to realize that VCUSM21P vaccine carries the genes that encode the non-toxic A (A1) subunit and the immuno-enhancing CTB molecules. The 7^th^ amino acid of the *ctx*A which is the arginine has been substituted with lysine (R7K), and the 112^th^ glutamate was substituted with glutamine (E112Q). In some vaccine construction designs, CT has been made to lack the ‘toxic’ ADP-ribosylating activity, by modifying the A1 subunit However, a loss of toxicity in VCUSM21P has been matched with a corresponding loss of its adjuvanticity, needing additional immunostimulants for vaccination [45]–[47]. Besides vibriocidal antibodies, IgA and IgG antibodies must have kept the animals free from developing cholera.

In future study, mutant strain of *hly*A and *rec* gene should be created to minimize the mild diarrhea and likelihood of in field recombination. Also immunological study could be extended by including the detection of anti-O specific polysaccharide responses, mucosal ASC responses, and responses to detect the induction of memory cells.

## Conclusion

In conclusion, we have expanded vaccine safety efforts and constructed a new cholera vaccine, VCUSM21P, against *V. cholerae* O139. This vaccine is suitably non-reactogenic and immunogenic *in vivo*, and protects animals from lethal *V. cholerae* O139 challenge. VCUSM21P could potentially help to reduce cholera disease burdens around the world leading to elimination of childhood mortality caused by *V. cholerae* O139 infection.
